# Influenza A Virus Does Not Encode a Tetherin Antagonist with Vpu-Like Activity and Induces IFN-Dependent Tetherin Expression in Infected Cells

**DOI:** 10.1371/journal.pone.0043337

**Published:** 2012-08-27

**Authors:** Michael Winkler, Stephanie Bertram, Kerstin Gnirß, Inga Nehlmeier, Ali Gawanbacht, Frank Kirchhoff, Christina Ehrhardt, Stephan Ludwig, Miriam Kiene, Anna-Sophie Moldenhauer, Ulrike Goedecke, Christina B. Karsten, Annika Kühl, Stefan Pöhlmann

**Affiliations:** 1 German Primate Center, Göttingen, Germany; 2 Institute of Virology, Hannover Medical School, Hannover, Germany; 3 Institute of Molecular Virology, Ulm University Medical Center, Ulm, Germany; 4 Institute of Molecular Virology, Westfälische-Wilhelms-University, Münster, Germany; 5 Institute for Cellular Chemistry, Hannover Medical School, Hannover, Germany; Lady Davis Institute for Medical Research, Canada

## Abstract

The interferon-induced host cell factor tetherin inhibits release of human immunodeficiency virus (HIV) from the plasma membrane of infected cells and is counteracted by the HIV-1 protein Vpu. Influenza A virus (FLUAV) also buds from the plasma membrane and is not inhibited by tetherin. Here, we investigated if FLUAV encodes a functional equivalent of Vpu for tetherin antagonism. We found that expression of the FLUAV protein NS1, which antagonizes the interferon (IFN) response, did not block the tetherin-mediated restriction of HIV release, which was rescued by Vpu. Similarly, tetherin-mediated inhibition of HIV release was not rescued by FLUAV infection. In contrast, FLUAV infection induced tetherin expression on target cells in an IFN-dependent manner. These results suggest that FLUAV escapes the antiviral effects of tetherin without encoding a tetherin antagonist with Vpu-like activity.

## Introduction

The interferon (IFN) system is an integral part of the innate defenses against viral infection [1]. Sensors of the IFN system detect the presence of viral components and trigger signaling cascades which result in the expression of IFN. Subsequently, binding of IFN to IFN receptors on the cell surface induces the expression of IFN-stimulated genes (ISGs), several of which have antiviral activity and protect the cell against invading viruses [1]. While important sensors and signal transducers of the IFN system have been identified, the nature of the ISGs responsible for the transition of cells into an antiviral state is less well defined.

Tetherin is a recently identified IFN-induced host cell protein, which was initially shown to restrict release of human immunodeficiency virus type 1 (HIV-1) particles from infected cells and to be counteracted by the HIV-1 protein Vpu [2], [3]. Subsequently, it was demonstrated that tetherin can also inhibit the cellular egress of other viruses and that several of these tetherin-sensitive viruses encode tetherin antagonists [4], [5], [6], [7], [8], [9], [10]. For instance, the release of Ebola virus (EBOV)-like particles, consisting solely of the viral matrix protein VP40, is inhibited by tetherin and coexpression of the EBOV glycoprotein (GP) counteracts this inhibition, thereby allowing unrestrained release of virus-like particles (VLPs) and potentially authentic EBOV from tetherin expressing cells [7], [8], [11], [12]. The presence of two membrane anchors in tetherin, an N-terminal transmembrane domain and a C-terminal GPI anchor, is critical for tetherin’s antiviral activity, since they allow tetherin to simultaneously insert into the viral and the cellular membrane, thereby forming a tether between virion and host cell [13].

Tetherin is localized in lipid rafts in the plasma membrane [14] and most viruses inhibited by tetherin use the plasma membrane to exit the host cell. Consequently, removal of tetherin from the plasma membrane is an important component of tetherin antagonism by the HIV-1 Vpu protein [15], [16]. In contrast, the EBOV-GP seems to inhibit tetherin without interfering with the localization of tetherin at the plasma membrane [7], [11], [12], [17] and the molecular mechanism underlying tetherin counteraction by this protein is at present unclear. Similarly, it remains elusive whether some viruses which use the plasma membrane as platform for budding are not targeted by tetherin.

Influenza A viruses (FLUAV), members of the orthomyxovirus family, cause annual epidemics and less frequently pandemics, which entail significant morbidity and mortality. Budding of FLUAV is driven by the viral membrane proteins hemagglutinin (HA), neuraminidase (NA), M2, an ion channel protein, and the viral matrix protein M1. The release of progeny particles occurs at the plasma membrane [18], [19], [20]. Yondola and coworkers previously showed that tetherin can inhibit release of VLPs driven by NA [21]. In addition, Watanabe and colleagues demonstrated that tetherin restricts release of FLUAV-VLPs from cells expressing most viral proteins but being devoid of NS1, while release of authentic FLUAV was not inhibited [22]. These observations suggest that authentic FLUAV might encode a tetherin antagonist, possibly NS1, which is not present in VLP systems.

Here, we demonstrate that tetherin does not efficiently inhibit release of FLUAV and we show that NS1 does not function as a tetherin antagonist. In fact, FLUAV infection of tetherin expressing cells did not compromise tetherin-dependent inhibition of the release of HIV-1 VLPs, indicating that none of the FLUAV proteins inhibits tetherin. Instead, FLUAV infection induced tetherin expression, indicating that FLUAV can ensure its release from tetherin positive cells by a novel mechanism.

## Results

### Tetherin Expression has Little Impact on Influenza A Virus Egress from Infected Cells

**Figure 1:**
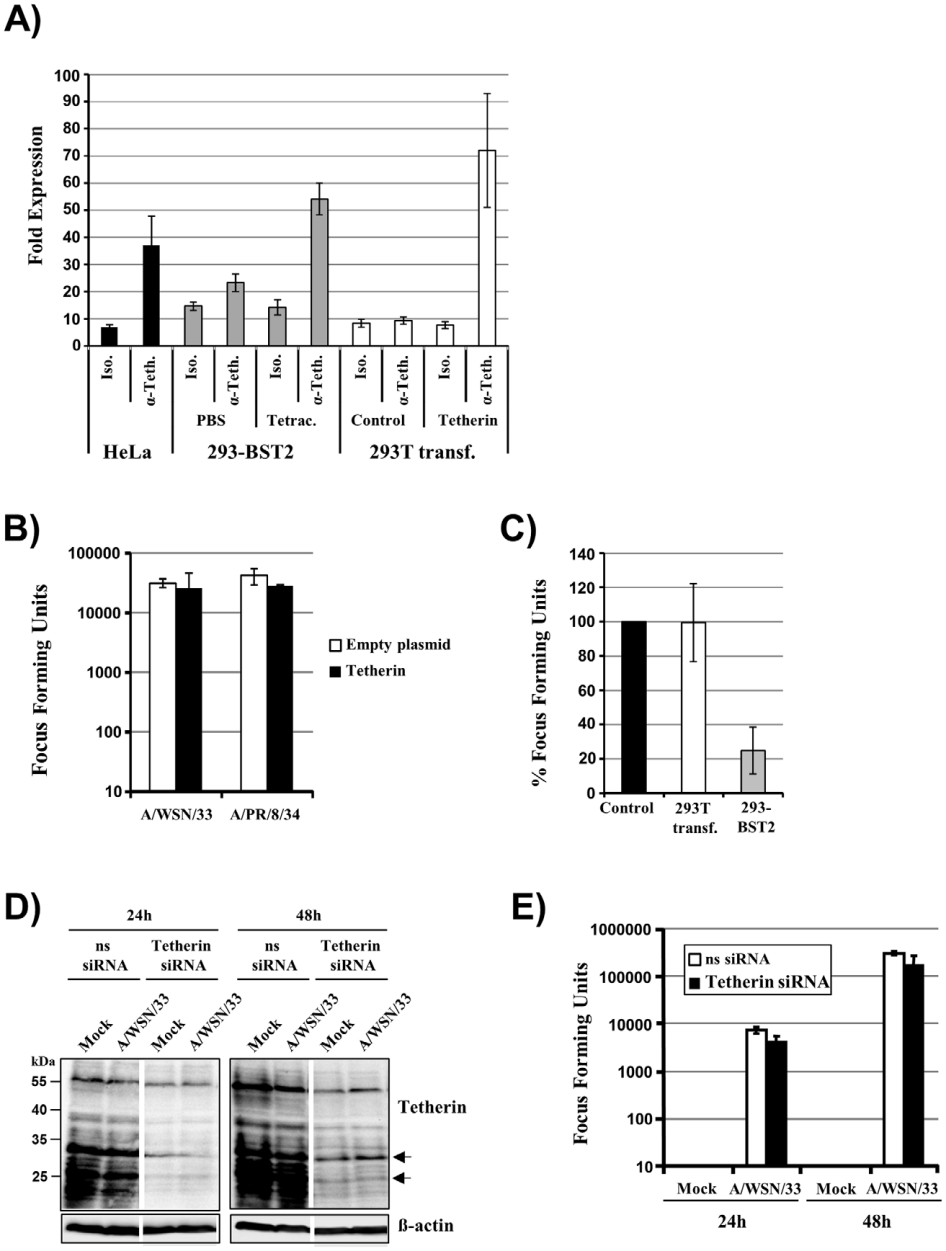
Tetherin has little impact on the release of infectious influenza A virus. (A) HeLa cells, tetracycline (Tetrac.) or PBS induced 293-BST2 cells and transfected 293T cells were stained with anti-tetherin or isotype-matched (Iso.) control antibody and staining was analyzed by FACS. The arithmetic mean channel fluorescence was measured and results are presented as fold increase compared to unstained cells. The average of six independent experiments is shown, error bars indicate standard error of the mean (SEM). (B) A tetherin expression plasmid or empty plasmid was transiently transfected into 293T cells and the cells were infected with the indicated FLUAV strains at an MOI of 1. After 60 min, the viral inocula were removed, the cells washed and incubated for 24 h with fresh medium. Thereafter, the infectivity in cell culture supernatants was determined employing the focus formation assay. The results of a representative experiment performed with triplicate samples are shown, error bars indicate standard deviation (SD). (C) The experiment was carried out as described in (B) but A/PR/8/34 release from PBS or tetracycline induced 293-BST2 cells and from empty plasmid or tetherin plasmid transfected 293T cells (293T transf.) was analyzed. The results represent the average of four independent experiments. Error bars indicate SEM. Release from PBS induced 293-BST2 cells or empty plasmid transfected 293T cells were set as 100% (control). (D–E) HeLa cells were transiently transfected with scrambled, non-sense (ns) siRNA or tetherin siRNA. At 24 h post transfection, the cells were infected with A/WSN/33 at a MOI of 1 or mock infected for 1 h at 37°C. Afterwards, cells were washed with PBS and tetherin expression in cell lysates was determined by Western blot. The results of single blots, from which irrelevant lanes were excised, are shown in panel (D). Tetherin signals are diffuse, due to heterogeneity in glycosylation with major signals in the range from 20–34 kDa (arrows indicate the major tetherin glycoforms). In parallel, the infectivity in the cell culture supernatants was determined employing the focus formation assay at 24 h and 48 h post infection. The results of a representative experiment performed with triplicate samples are shown in panel (E), error bars indicate SD. Similar results were obtained in a separate experiment.

We examined whether tetherin inhibits release of FLUAV from 293T cells transfected to express tetherin, 293 cells expressing tetherin upon induction with tetracycline (293-BST2) and HeLa cells expressing endogenous tetherin. FACS analysis demonstrated robust expression of tetherin on these cell types, with transfected 293T cells harboring the highest levels of tetherin, followed by tetracycline-induced 293 cells and HeLa cells (Fig. 1A). Transfection of 293T cells with a tetherin expression plasmid imposes an efficient restriction on the release of vpu-defective HIV-1 [11], [23]. In contrast, ectopic expression of tetherin did not modulate release of FLUAV A/WSN/33 and A/PR/8/34, as determined by the quantification of infectious units in culture supernatants by focus formation assay (Fig. 1B, C). A roughly 4-fold reduction of A/PR/8/34 release was observed upon induction of tetherin expression on 293-BST2 cells (Fig. 1C), potentially because of the more uniform tetherin expression on these cells compared to transfected 293T cells (n ot shown). However, this effect was not statistically significant (p = 0.153). Finally, HeLa cells were examined, which express sufficient amounts of endogenous tetherin to inhibit HIV-1 release [23]. Transfection of HeLa cells with tetherin specific siRNA efficiently reduced tetherin levels while a scrambled, non-sense siRNA had no effect (Fig. 1D). Infection of the siRNA transfected HeLa cells with A/WSN/33 or A/PR/8/34 and quantification of the number of infectious virions released into the supernatants showed no appreciable difference between tetherin knock-down and control cells (Fig. 1E and data not shown), indicating that endogenous tetherin does not restrict release of infectious FLUAV. These results indicate that FLUAV release is not efficiently inhibited by tetherin, in accordance with published work [22], [24], although a modest blockade of FLUAV release can be observed upon engineered expression of high levels of tetherin.

### NS1 does not Function as a Vpu-like Tetherin Antagonist

**Figure 2:**
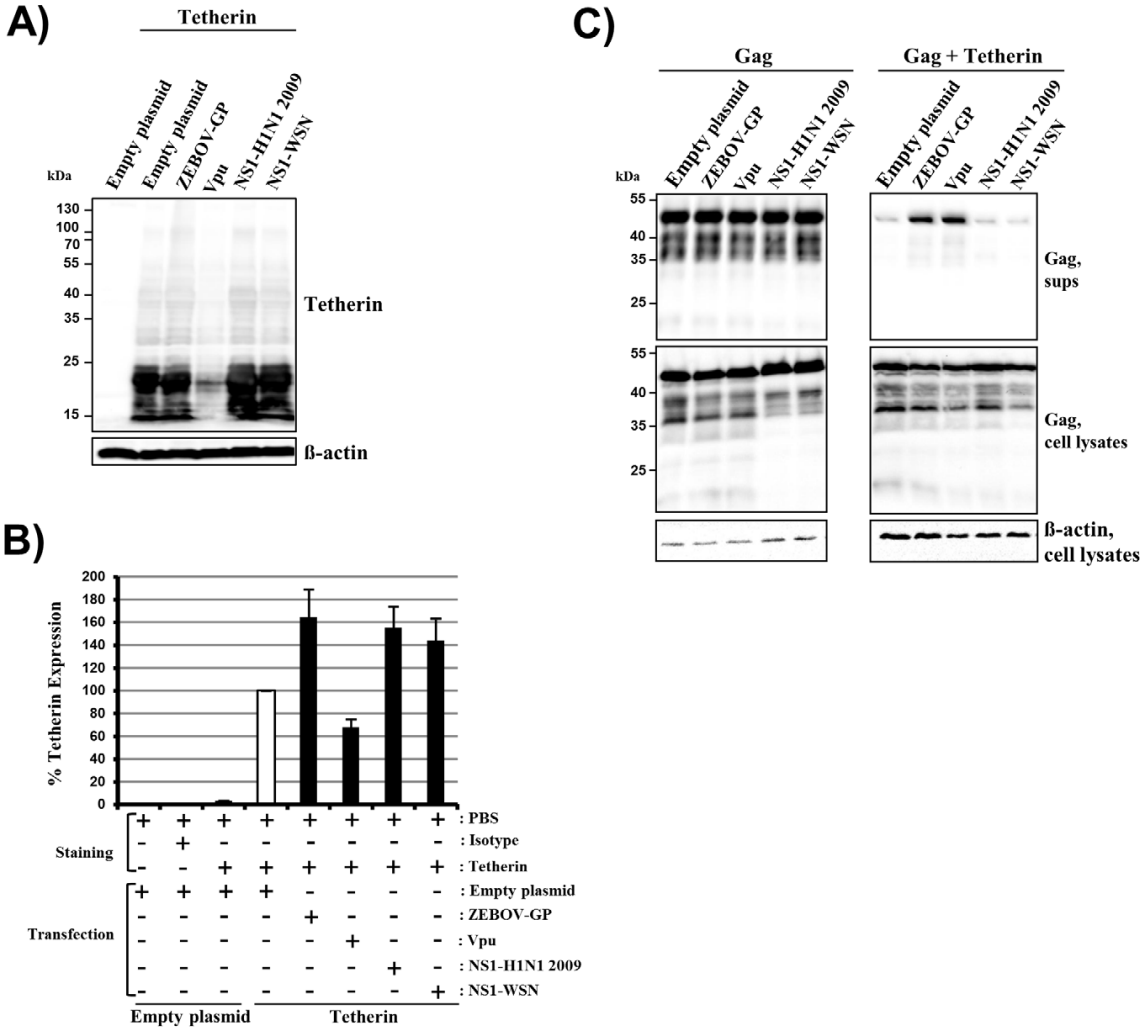
NS1 does not antagonize tetherin. (A) Plasmids encoding the indicated proteins or empty plasmid were transfected into 293T cells and expression of tetherin and β-actin in cell lysates was determined by Western blot. Similar results were obtained in two separate experiments. (B) The experiment was performed as described in (A) but cell surface expression of tetherin was determined by FACS. The average of 6 to 8 independent experiments is shown, error bars indicate standard error of the mean (SEM). (C) Plasmids encoding HIV-1 Gag, tetherin and the indicated viral proteins were cotransfected into 293T cells and the presence of Gag in cell lysates and cell culture supernatants was determined by Western blot. Expression of β-actin in cell lysates was assessed as loading control. The results were confirmed in three separate experiments.

A simple explanation for the lack of antiviral activity of tetherin against FLUAV would be that the virus encodes a tetherin antagonist, like the Vpu protein of HIV-1. The NS1 protein was a potential candidate for a tetherin antagonist, since this protein was not included in a FLUAV-based VLP system, which was previously shown to generate particles susceptible to inhibition by tetherin [22]. In order to investigate if NS1 is a tetherin antagonist, we first analyzed if this protein, like HIV-1 Vpu, interferes with the expression of tetherin. Coexpression of Vpu reduced tetherin levels within cell lysates (Fig. 2A) and at the cell surface (Fig. 2B) compared to cells transfected with empty plasmid. In contrast, the presence of EBOV-GP did not diminish tetherin expression, as expected [11], [12]. In fact, the expression of tetherin on cells cotransfected with EBOV-GP expression plasmid was higher compared to cells transfected with empty plasmid, an effect we had previously observed and shown to be due to unspecific interference of empty plasmid with tetherin surface levels [11]. Coexpression of the NS1 proteins from FLUAV A/HH/2009/04 or A/WSN/33 did not decrease tetherin levels at the cell surface or within cell lysates (Fig. 2A, B), despite efficient expression of these proteins in 293T cells (Fig. S1), demonstrating that FLUAV NS1, unlike HIV-1 Vpu, does not interfere with tetherin expression and cell surface levels.

The EBOV-GP antagonizes tetherin without modulating total tetherin expression or tetherin presence at the cell surface [11], [12]. We therefore analyzed if NS1 counteracts tetherin-mediated inhibition of the release of HIV-1 p55 Gag-based VLPs from transfected cells. The expression of tetherin markedly reduced the release of Gag into culture supernatants compared to cells transfected with empty plasmid and this effect was rescued by coexpression of Vpu or EBOV-GP (Fig. 2C), in agreement with previous studies [7], [11], [12]. In contrast, expression of NS1 did not relieve the block on HIV-1 VLP release imposed by tetherin (Fig. 2C), indicating that NS1 does not antagonize tetherin, at least under the conditions tested here.

### Evidence that Influenza A Virus does not Encode a Tetherin Antagonist with Vpu-like Activity

**Figure 3:**
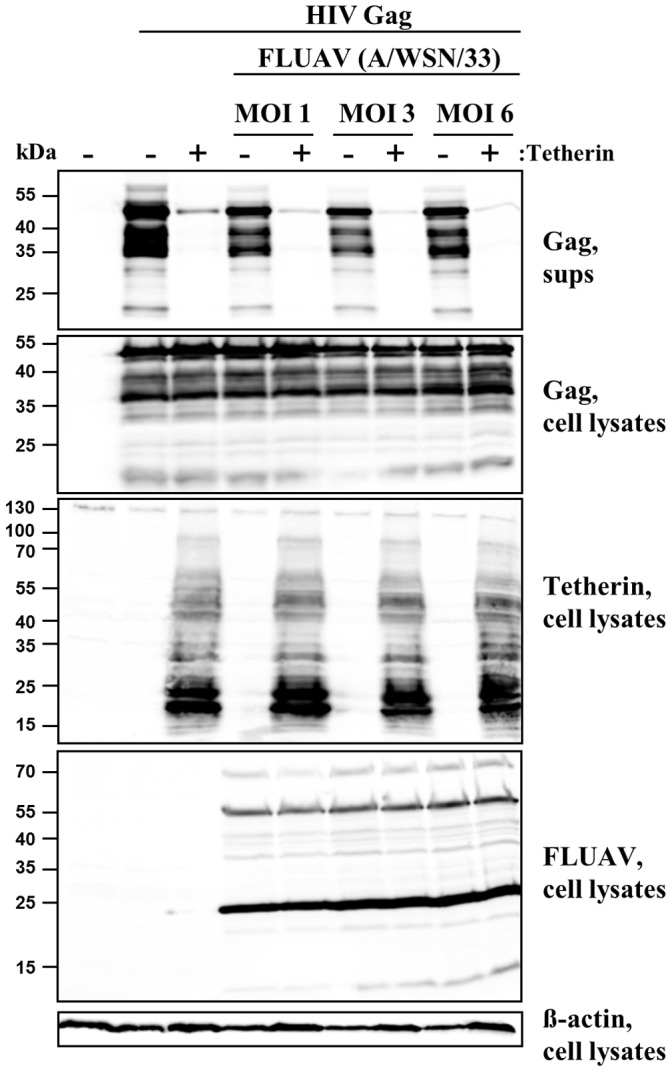
Influenza A virus infection does not antagonize tetherin. Plasmids encoding HIV-1 Gag and tetherin were cotransfected into 293T cells and the cells were subsequently infected with A/WSN/33 at the indicated MOIs or mock infected. At 24 h post infection the presence of Gag in cell lysates and culture supernatants (sups) as well as the expression of tetherin, FLUAV antigens and β-actin in cell lysates was determined by Western blot. Similar results were obtained in four separate experiments conducted with MOIs of 0.03 and 0.3.

The inability of NS1 to counteract tetherin does not exclude the possibility that other FLUAV proteins antagonize tetherin or that NS1 inhibits tetherin in the presence of other FLUAV proteins. To investigate this scenario, we examined the effect of FLUAV infection on the tetherin-imposed restriction of HIV-1 VLP release. For this, HIV-1 p55 Gag was expressed in the presence and absence of tetherin followed by infection with escalating doses of A/WSN/33 and subsequent determination of Gag release into the culture supernatants. The expression of tetherin markedly reduced the release of Gag into the supernatants but had no effect on total Gag expression in cell lysates (Fig. 3). Notably, the tetherin-mediated restriction of Gag release was not rescued by A/WSN/33 infection (Fig. 3), indicating that FLUAV does not encode a tetherin antagonist with Vpu-like activity.

### Influenza A Virus Infection Induces Tetherin Expression at the Cell Surface

**Figure 4:**
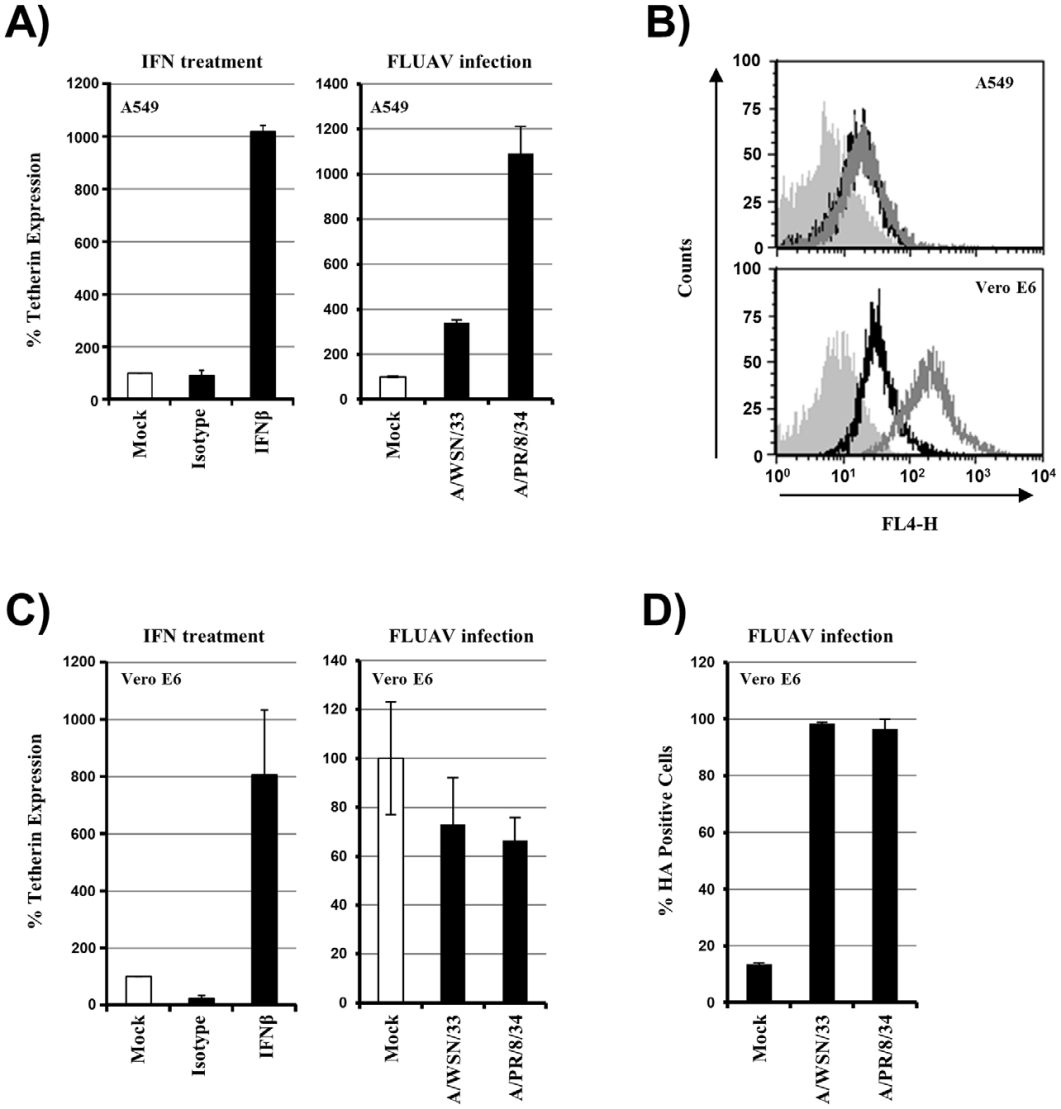
Influenza A virus infection induces tetherin expression at the cell surface in an interferon-dependent manner. (A) A549 cells were treated with 10,000 U/ml IFNβ (left panel) or infected with the indicated viruses at an MOI of 1 (right panel). After 24 h, the expression of tetherin at the cell surface was determined by FACS. The average of two separate experiments is shown, error bars indicate SEM. Similar results were obtained in two separate experiments. (B) A549 and Vero E6 cells, which were not exposed to FLUAV or IFN, were mock treated (filled grey histogram) or incubated with isotype matched control antibody and secondary antibody (black line) or incubated with anti-tetherin antibody and secondary antibody (grey line). Subsequently, staining was analyzed by FACS. (C) The experiment was carried out as described in (A) but Vero E6 cells were used. The average ± SEM of two separate experiments is shown for IFN induction of tetherin expression (left panel), while the average ± SD of a single experiment performed in triplicates are shown for FLUAV infection. Similar results were obtained in a separate experiment. (D) Expression of hemagglutinin (HA) on the cells analyzed in (C) was determined by FACS. The average ± SEM of two separate experiments is shown.

Since tetherin did not inhibit FLUAV release, we finally asked whether FLUAV infection triggers tetherin expression. Indeed, A/PR/8/34 infection or IFNβ treatment of A549 cells, which have an intact IFN system, induced robust expression of tetherin at the cell surface, while tetherin was not detected on uninfected cells (Fig. 4A, B). Similarly, infection of A549 cells by A/WSN/33 increased tetherin expression, although not to the same level as A/PR/8/34 infection. In contrast, a marked upregulation of tetherin levels on Vero E6 cells, which express endogenous tetherin (Fig. 4B), was only observed upon IFNβ treatment but not A/PR/8/34 or A/WSN/33 infection (Fig. 4C, D), in accordance with the published finding that Vero E6 cells are defective in the synthesis of IFN [25]. Instead, FLUAV infection slightly reduced cell surface tetherin levels in Vero E6 cells. Thus, FLUAV infection can trigger tetherin expression at the surface of susceptible cells containing a functional IFN system.

## Discussion

Tetherin is an IFN-induced protein which can block the release of different enveloped viruses from infected cells by forming a physical tether between the cellular and the viral membrane [26], [27]. Several enveloped viruses which are targeted by tetherin encode for tetherin antagonists, which allow for efficient viral spread in tetherin expressing target cells [26], [27]. Understanding how viruses escape tetherin’s antiviral function might provide important insights into virus host cell interactions and reveal novel targets for antiviral intervention. Here, we show that FLUAV escapes tetherin’s antiviral activity apparently without encoding a tetherin antagonist and even induces tetherin expression in an IFN-dependent manner.

Tetherin was reported to inhibit the release of retro-, filo-, arena- and herpes viruses or virus-like particles and, with the exception of arenaviruses, tetherin antagonists have been identified in all of the afore mentioned viruses [26], [27]. Thus, tetherin seems to exert potent antiviral activity in the infected host which forced viruses to acquire antagonists to overcome this antiviral defense. A joint feature of most viruses targeted by tetherin is their usage of the plasma membrane as platform for the budding of progeny particles and tetherin has been shown to localize to lipid rafts in the plasma membrane [14]. Budding of FLUAV is driven by the membrane proteins HA, NA and M2 and the matrix protein M1 and also occurs at the plasma membrane [18], [19], [20], making FLUAV a potential target for tetherin. However, the tetracycline-induced expression of high levels of tetherin only modestly reduced FLUAV release from 293 cells and transient expression of tetherin did not interfere with release of A/WSN/33 or A/PR/8/34. Similarly, knock-down of endogenous tetherin in HeLa cells did not augment the presence of infectious FLUAV in culture supernatants, suggesting that tetherin has only a minor, if any, effect on FLUAV release, in accordance with published data [22], [24]. Trypsin can counteract tetherin’s antiviral activity [28] and was present, with one exception (below), in all experiments for either 1 h, during the incubation of target cells with infection medium, or during the entire experiment (24 h) in order to ensure efficient influenza virus activation. However, identical results were obtained for FLUAV release independent of trypsin being present for 1 h or during the entire experiment (Fig. 1B and 1C) and, more importantly, trypsin failed to counteract the tetherin-dependent inhibition of the release of HIV-1-based VLPs (data not shown), potentially because the amount of trypsin used for all experiments was lower than that employed by the previous study. Finally, the experiment shown in Fig. 3 was conducted entirely in the absence of exogenous trypsin, because the neuraminidase of the A/WSN/33 virus can recruit plasminogen present in fetal calf serum to ensure HA activation [29]. Thus, trypsin did not compromise tetherin’s antiviral activity under the conditions chosen in the present study. Therefore, the efficient release of FLUAV from tetherin expressing cells suggests that the virus might encode a tetherin antagonist - a possibility addressed by the present study. Alternatively, FLUAV might have evolved a different strategy to avoid the presence of tetherin at viral budding sites and/or the insertion of tetherin in the viral membrane.

The HIV-1 Vpu protein reduces the plasma membrane localization of tetherin and induces tetherin degradation in lyso- or proteasomes [15], [16], [30]. Although the ability of Vpu to deplete tetherin can be dispensable for its anti-tetherin activity [31], these findings suggest that Vpu mainly counters tetherin by removing it from the cellular location where it exerts its antiviral activity, the plasma membrane. In contrast, NS1 did not decrease tetherin levels at the plasma membrane or total expression of tetherin; in fact, NS1 slightly increased total tetherin levels. The EBOV-GP, another tetherin antagonist, has previously been found to antagonize tetherin without interfering with tetherin expression and presence at the plasma membrane [8], [11], [12], suggesting that viral antagonists may block tetherin’s antiviral activity without changing the presence of tetherin at the site of viral budding. If NS1 employs such a strategy to antagonize tetherin, expression of this protein should rescue the tetherin-induced blockade of HIV-1 VLP release. However, NS1 was inactive. Furthermore, NS1 might exert anti-tetherin activity only in the presence of other FLUAV proteins or only in the context of FLUAV and not HIV-1 infection, but a recent study with NS1-defective FLUAV argues against this possibility [24]. Finally, viral proteins other than NS1 might function as tetherin antagonists. If this assumption was correct, release of HIV-1 VLPs should be rescued by FLUAV infection of Gag producing cells. We tested this hypothesis by infecting HIV-1 Gag transfected 293T cells with A/WSN/33 at a multiplicity of infection (MOI) of 1, 3 and 6 but did not observe any counteraction of the tetherin-imposed restriction to Gag release. Collectively, these results suggest that FLUAV does not encode a tetherin antagonist with Vpu- or EBOV-GP-like activity.

Tetherin expression can be induced by type I IFN, although several cells and tissues, including lung, seem to express tetherin constitutively [32]. FLUAV infection induces IFN production which is negatively regulated but not abrogated by the viral IFN antagonist NS1 [33], suggesting that infection could induce tetherin expression. Examination of the human lung adenocarcinoma epithelial cell line A549 revealed a strong upregulation of tetherin cell surface expression upon treatment with IFNβ (10-fold) and upon infection by A/PR/8/34 (11-fold) and, to a lesser degree by A/WSN/33 (3-fold). In contrast, FLUAV infection of Vero E6 cells, which have a well characterized defect in the IFN system [25], [34], did not result in augmented tetherin expression at the cell surface. These findings, jointly with the observations that cellular supernatants depleted from FLUAV particles did not trigger tetherin expression (not shown), suggest that IFN production induced by FLUAV infection leads to the expression of cell surface tetherin. Could the IFN-induced or constitutively expressed tetherin modulate FLUAV spread in the host? One can speculate that tetherin may not only fail to interfere with viral release but may actually promote viral spread, since tetherin has been shown to negatively regulate IFN production by plasmacytoid dendritic cells via interactions with ILT7 [35].

In sum, our results demonstrate that FLUAV infection induces tetherin expression in an IFN-dependent manner and that tetherin does not efficiently inhibit viral egress, although FLUAV lacks a tetherin antagonist with Vpu- or EBOV-GP-like activity. Why does tetherin fail to inhibit FLUAV with appreciable efficiency? HIV-1 and FLUAV employ different plasma membrane microdomains for budding and it is conceivable that tetherin is present at budding sites of HIV-1 but not FLUAV [36]. However, a recent study by Bruce and colleagues suggests that HA and tetherin colocalize to a substantial extent at the surface of FLUAV infected cells [24]. This observation, jointly with the findings that tetherin is incorporated into HIV-1 but not FLUAV particles [22], [24], suggests that FLUAV prevents insertion of tetherin into the viral envelope and is thus immune to tetherin’s antiviral action. Whether the exclusion of tetherin from virions is due to general structural constraints of the FLUAV budding process or if one or more viral proteins specifically exclude tetherin from FLUAV but not HIV-1 particles remains to be determined.

During the revision of the present manuscript findings by Mangeat and colleagues on the role of tetherin in FLUAV infection were reported [37]. This study found that exogenous and endogenous expression of tetherin markedly diminished the release of FLUAV and electron microscopic evidence for retention of FLUAV at the surface of tetherin expressing cells was obtained [37]. These findings largely contrast the results reported by Watanabe and colleagues [22], Bruce and colleagues [24] and the findings reported in the present manuscript. The reasons for these discrepant observations are not apparent, although differences in the experimental conditions employed, for instance the efficiency of tetherin expression, might play a role. Further studies are thus required to clarify the impact of tetherin on FLUAV infection. These endeavors should comprise quantification of tetherin copy numbers on cell lines and primary cells used for FLUAV and HIV infection and correlation of the expression levels with antiviral activity – or absence thereof.

## Materials and Methods

### Plasmids

For the construction of expression plasmids for NS1 from FLUAV A/WSN/33 and A/HH/2009/04, the NS1 sequences were amplified from plasmids of the respective 8 plasmid systems using the primer pairs WSN-NS1-5Acc 5-GGGGGTACCACCATGGATCCAAACACTGTGTCAAGC-3 (forward), WSN-NS1-3Xho 5-GCGCTCGAGTCAAACTTCTGACCTAATTGTTCC-3 (reverse), swi09-NS1-5Acc 5-GGGGGTACCACCATGGACTCCAACACCATGTCAAGC-3 (forward) and swi09-NS1-3Sal 5-GCGGTCGACGGATCCTCATTTCTGCTCTGGAGGTAGTGAAG-3 (reverse). Subsequently, the PCR products were cloned into pCAGGS using the Acc65I and XhoI restriction sites. The integrity of all clones was verified by sequencing. Plasmids encoding HIV-1 Gag (p55) [38], Vpu [39], ZEBOVGP [40] and human tetherin [41] were described previously.

### Cell Culture

We used Dulbecco’s modified Eagle’s medium (DMEM; Invitrogen) for culture of 293-BST2 [11], [42], 293T (ATCC no. CRL-11268), Vero E6 (ATCC no. CRL-1568) and A549 (ATCC no. CCL-185) cells, and minimum essential medium (MEM; PAA) for culture of MDCK II cells (ATCC no. CRL-2936). All media were supplemented with 10% fetal calf serum (FCS), penicillin and streptomycin, and all cell cultures were maintained at 37°C under a 5% CO_2_ atmosphere.

### Virus-like Particles

293T cells were seeded at a density of 2.6×10^5^ cells/well in 6-well plates 24 h prior to transfection. At the following day, the cells were cotransfected with plasmids encoding HIV-1 Gag and tetherin or empty plasmid in combination with plasmids encoding ZEBOV-GP, Vpu or NS1 or empty plasmid at a ratio of 2∶1:1, reaching a total DNA-concentration of 6 µg per well. Two days after transfection, supernatants were harvested and centrifuged at 4000 rounds per minute (rpm) for 5 min to remove cellular debris. Subsequently, supernatants were loaded onto 20% sucrose and centrifuged at 17,000 g for at least 4 h at 4°C. The supernatants of the centrifugation reactions were removed and the pellets resuspended in SDS-loading buffer. For production of cell lysates, the cells were washed with 1xPBS and lysed with 150–200 µL SDS-loading buffer. All samples were denatured for 30 min at 95°C, separated by SDS-PAGE and proteins detected by immunoblotting.

### Production of Influenza Virus

Influenza virus was produced in hens’ eggs as described [43]. In brief, pathogen-free embryonated hens’ eggs were inoculated with virus dilutions via the allantoic sac. After incubation of eggs for 48 h at 37°C, the allantoic fluids were collected, tested for hemagglutinating activity, pooled, aliquoted, and stored at −70°C. Alternatively, T-75 flasks of MDCK cells (80–90% confluence) were washed with PBS and inoculated with 2 mL MEM (supplemented with 1 µg/mL TPCK-Trypsin and 0.2% BSA) containing 1×10^6^ focus forming units (FFU) influenza virus. After an incubation period of 1 h at 37°C, 10 ml MEM (see above) without virus was added and the cells were further incubated at 37°C. At 24 h post-infection, the supernatant was harvested, passed through a 0.45 µm-pore-size filter, aliquoted, and stored at −80°C. The virus titer was determined by the focus formation assay.

### Virus Titration by Focus Formation Assay

Virus titers were determined in a focus formation assay essentially as described [43], [44]. MDCK II target cells were seeded in 96 well culture plates at a density of 6×10^4^/well and incubated at 37°C under 5% CO_2_ for 24 h. On the following day, supernatants from the infected cultures were collected, 10-fold serially diluted in MEM containing 0.1% BSA and 1 µg/mL TPCK*-*Trypsin (Sigma) and 50 µL of each dilution inoculated onto confluent monolayers of MDCK II cells in 96-well culture plates. The infected cells were incubated at 37°C and 5% CO_2_ for 1 h with shaking at 20 min intervals. Subsequently, inoculates were removed and replaced with 100 µL of a 1% Avicel overlay containing 0.1% BSA and 2 µg/mL TPCK-Trypsin. After an incubation at 37°C, 5% CO_2_ for 24 h, the cells were washed twice with PBS and were then fixed with 4% formalin in PBS (100 µL/well) for 10 min at room temperature. The formalin was removed and the cells were washed two times with PBS. After incubation with 100 µL/well Quencher (0.5% Triton X-100, 20 mM glycine in PBS) for 10 min, the cells were washed with wash buffer (WB) (0.1% Tween 20 in PBS) and then blocked with 50 µL of blocking buffer (BB) (0.5% Tween, 20% BSA in PBS) at 37°C under 5% CO_2_ for 30 min. A polyclonal goat antibody raised against influenza A virus (Millipore) served as primary antibody and HRP-conjugated anti-goat antibody (KPL) was used as secondary antibody; both were diluted 1∶1,000 in BB. Cells were incubated at 37°C and 5% CO_2_ for 30 min with 50 µL of the primary antibody. After a triple wash with WB, the cells were incubated with 50 µL of the secondary antibody for 30 min. Finally, the cells were washed again and incubated with 50 µL of the HRP-substrate (True Blue; KPL) until blue spots became visible. Foci were enumerated and viral titers calculated as focus-forming units (FFU) per mL.

### Infection Experiments with Influenza Viruses

For infection experiments with FLUAV, 293T cells were seeded in 6-well plates at a density of 2.5×10^5^ cells/well, transiently transfected with plasmids encoding tetherin or empty plasmid (routinely, transfection efficiencies of 75% and higher were reached), washed and inoculated with A/WSN/33 and A/PR/8/34 virus (diluted in minimum essential medium (MEM) supplemented with 0.2% bovine serum albumin [BSA]) at the indicated MOI. Alternatively, A549, Vero E6 or HeLa cells were seeded in 6-well plates at a density of 2.5×10^5^ cells/well and either washed and infected at 24 h after seeding (A549, Vero E6) as described above for 293T cells or Lipofectamine (Invitrogen) transfected (HeLa) with 100 pmol siRNA/well and infected at 24 h post transfection. Viruses were allowed to bind to target cells for 1 h at 37°C in the presence of 1 µg/mL TPCK-trypsin, with the exception of the experiment shown in Fig. 3, which was conducted without addition of exogenous trypsin. Subsequently, the infection medium was removed, MEM supplemented with 0.2% BSA and 1 µg/mL TPCK-trypsin or PBS was added and the cells were incubated for 24 h. Thereafter, the cell culture supernatants were collected, stored at −80°C and the number of infectious virus particles in the supernatants was quantified. Additionally, cells were collected and processed for FACS analysis and immunoblot.

### Flow Cytometry

For analysis of surface expressed proteins, cells were detached washed and stained. For detection of tetherin surface expression we used the previously described monoclonal antibody HM1.24 [45] or anti-CD317 (BioLegend). Isotype-matched controls were from R&D Systems. For detection of FLUAV infection we used a polyclonal goat antibody raised against complete FLUAV (Chemicon). After binding of primary antibodies for 30 min at 4°C, cells were washed twice and incubated for 30 min at 4°C with DyLight 649-coupled anti-mouse or Cy5-coupled anti-goat secondary antibodies (Dianova). After two final washing steps cells were fixated with 2% PFA and analyzed in a Becton Dickinson LSR II flow cytometer.

### Immunoblot

For immunoblotting, lysed VLP/virus preparations and cell lysates were separated by SDS-PAGE and transferred onto nitrocellulose membranes. An anti-p24 hybridoma supernatant (183-H12-5C) [46] was used at a dilution of 1∶500 for detection of p55-Gag in supernatants and cell lysates. Expression of FLUAV proteins in infected cells was analyzed by using a polyclonal goat anti-FLUAV antibody (Chemicon) at a dilution of 1∶1,000. Tetherin expression in cell lysates was detected with a rabbit anti-human tetherin antibody (NIH AIDS Research and Rerference Reagent Program, #11721) at a dilution of 1∶1,000. For loading control, the stripped membranes were incubated with a monoclonal mouse anti-ß-actin antibody (Sigma) at a dilution of 1∶1,000. For detection of bound antibodies, horseradish peroxidase-(HRP-)-labelled antibodies (Dianova) with the appropriate species specificity were used at a dilution of 1∶5,000.

## Supporting Information

Figure S1 NS1 proteins are expressed in transfected 293T cells. Expression plasmids for NS1 from A/WSN/33 and A/HH/2009/04 were transiently transfected into 293T cells and protein expression was detected with an antibody raised against NS1 protein from A/California/06/2009 (H1N1). Please note that the reduced signal of NS1-WSN compared to NS1-H1N1 2009 is most likely due to the latter protein being more similar to the antigen against which the antibody was raised.
(TIF)
