# Supplementary figures and images for: Influenza A Virus Does Not Encode a Tetherin Antagonist with Vpu-Like Activity and Induces IFN-Dependent Tetherin Expression in Infected Cells
Source: PLoS One. 2012 Aug 27;7(8):e43337. doi: 10.1371/journal.pone.0043337 (PMC3428345; doi:10.1371/journal.pone.0043337)

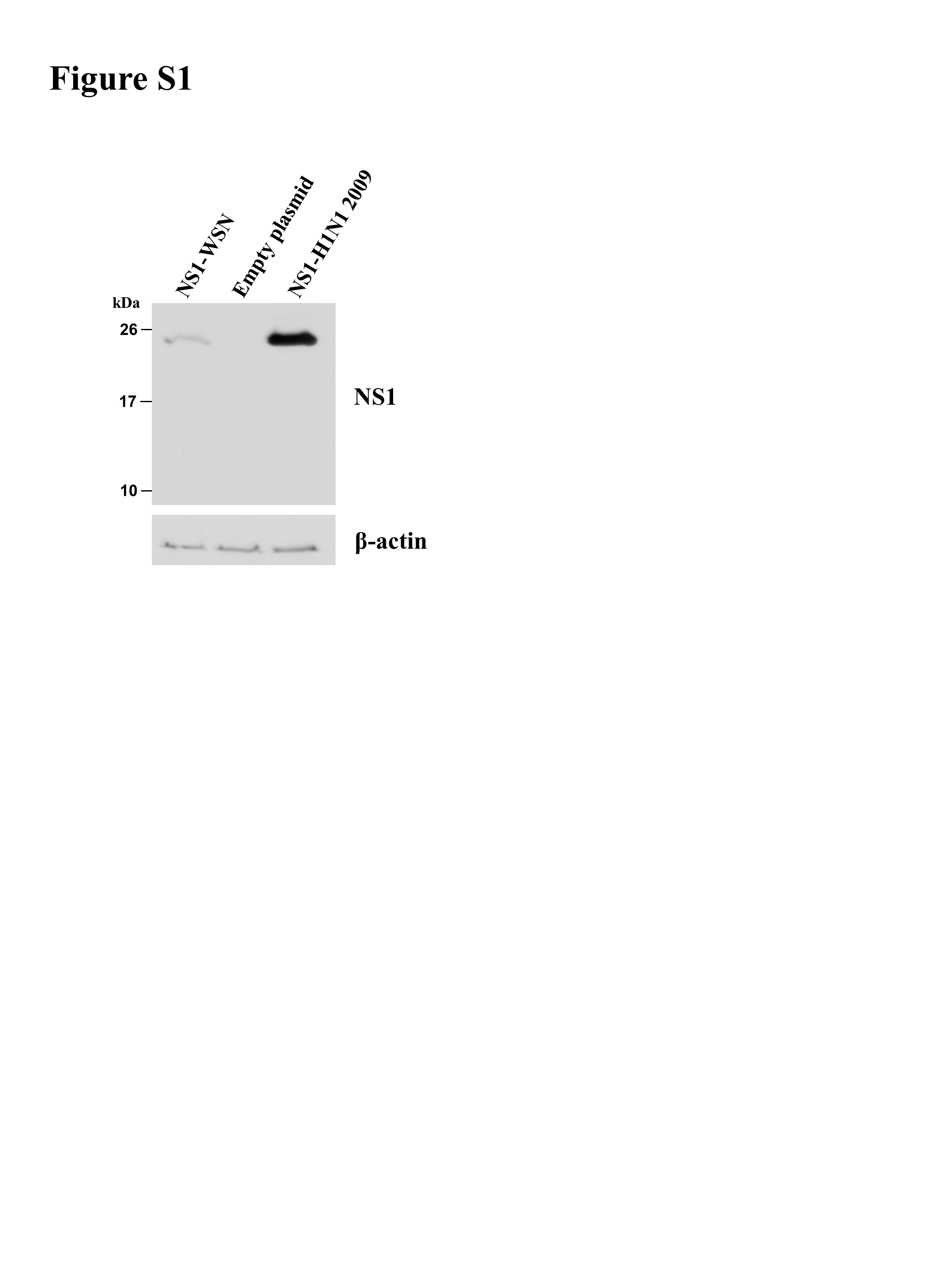

Supplement: Figure S1 — NS1 proteins are expressed in transfected 293T cells. Expression plasmids for NS1 from A/WSN/33 and A/HH/2009/04 were transiently transfected into 293T cells and protein expression was detected with an antibody raised against NS1 protein from A/California/06/2009 (H1N1). Please note that the reduced signal of NS1-WSN compared to NS1-H1N1 2009 is most likely due to the latter protein being more similar to the antigen against which the antibody was raised. (TIF) [file pone.0043337.s001.tif]
